# Ultra-Bright and -Stable Red and Near-Infrared Squaraine Fluorophores for *In Vivo* Two-Photon Imaging

**DOI:** 10.1371/journal.pone.0051980

**Published:** 2012-12-14

**Authors:** Kaspar Podgorski, Ewald Terpetschnig, Oleksii P. Klochko, Olena M. Obukhova, Kurt Haas

**Affiliations:** 1 Department of Cellular and Physiological Sciences and the Brain Research Centre, University of British Columbia, Vancouver, British Columbia, Canada; 2 SETA BioMedicals, Urbana, Illinois, United States of America; 3 State Scientific Institution “Institute for Single Crystals” of National Academy of Sciences of Ukraine, Kharkiv, Ukraine; Instituto de Neurociencias de Alicante UMH-CSIC, Spain

## Abstract

Fluorescent dyes that are bright, stable, small, and biocompatible are needed for high-sensitivity two-photon imaging, but the combination of these traits has been elusive. We identified a class of squaraine derivatives with large two-photon action cross-sections (up to 10,000 GM) at near-infrared wavelengths critical for *in vivo* imaging. We demonstrate the biocompatibility and stability of a red-emitting squaraine-rotaxane (SeTau-647) by imaging dye-filled neurons *in vivo* over 5 days, and utility for sensitive subcellular imaging by synthesizing a specific peptide-conjugate label for the synaptic protein PSD-95.

## Introduction

Since its inception [1], two-photon excitation microscopy (TPM) has been a powerful tool in cell and systems biology. In TPM, fluorophores are excited by the simultaneous absorption of two photons, each with lower energy than that required for single-photon excitation. TPM imaging has high resolution because two-photon absorption is proportional to the squared incident light intensity, confining excitation to a small volume near the focal point. TPM is particularly suited for *in vivo* imaging [2]–[4], since tissues are transparent to the red-shifted excitation wavelengths used, and photodamage is reduced due to the limited excitation volume [5].

The need for better, biocompatible fluorophores with large two-photon cross-sections for high sensitivity *in vivo* imaging has previously been identified [6]–[8]. The strongest limitations on imaging depth [9] and signal rate [10] in TPM have been photodamage and/or background fluorescence due to high excitation powers required to adequately excite fluorophores. Brighter dyes would allow imaging deeper into tissues, at higher rates, with lower excitation power. An ideal fluorophore for sensitive *in vivo* two-photon imaging should show the following characteristics: 1) Strong two-photon absorption in the near-infrared (NIR) window of wavelengths, between 750 and 950 nm, where absorption and scattering by tissues is minimized 2) High fluorescence quantum yield 3) High photostability, to ensure that each molecule can be excited many times before bleaching 4) High chemical stability within cells 5) No toxicity or other side effects to cells.

The brightest organic dyes and proteins commonly used for *in vivo* two-photon imaging, such as Rhodamine B and eGFP, have peak two-photon action cross-sections of 200GM in the NIR window. Quantum dots and other nanocrystals show stronger absorption, with action cross-sections as large as 47,000GM, and can be excited repeatedly without photobleaching [6]. However, quantum dots are considerably larger than organic dyes, and require appropriate coatings, which can be bulky, to reduce cytotoxicity and aggregation [7], [11]. Larger fluorophores may be problematic for studies tracking the motion of tagged biomolecules, such as fluorescence correlation spectroscopy [12] and single-molecule imaging [13], due to potential effects on protein diffusion, localization, and interactions. There has consequently been interest in developing smaller probes with brightness and stability similar to existing quantum dots [14].

## Results

**Figure 1:**
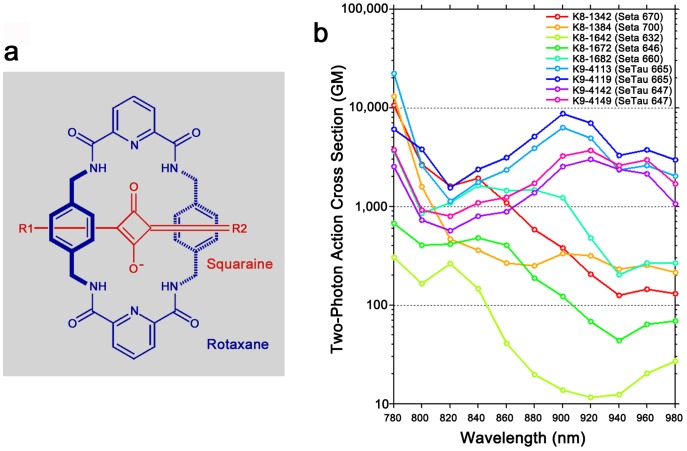
Squaraine derivatives with large two-photon action cross-sections in the NIR window. **a)** Structure of squaraine-rotaxanes. Squaraines contain a characteristic squarylium core flanked by nucleophilic motifs, forming an electron Donor-Acceptor-Donor structure. The macrocyclic ‘cage’ sterically shields the more reactive squarylium core, increasing its stability. **b)** Two-photon action cross-sections of squaraine derivatives. Dye names with a prefix of K8 denote squaraines, K9 prefixes denote squaraine-rotaxanes. Cross-sections were obtained by ratiometric imaging and calculated using emission spectra measured from BSA-conjugate (K8-1342), IgG-conjugate (K8-1384), or free dye (all others).

Squaraine dyes are a class of red/NIR fluorophores produced by a condensation reaction of electron-rich molecules with squaric acid, a small dibasic acid with a unique square carbon backbone (Fig. 1a). The donor-acceptor-donor structure of squaraines is conducive to strong two-photon absorption [15], and squaraines have been identified with two-photon action cross-sections as large as 33,000GM [16], but with molecular weights orders of magnitude smaller than quantum dots. The remarkable properties of squaraines have received attention for applications in photoconductivity, solar cells, and nonlinear optics [17]–[19]. Squaraine-based fluorescent sensors have been developed for a variety of analytes including Ca^2+^ [20], pH [21], protein and DNA, and squaraine-based labels exhibit an increase in fluorescence intensity and lifetime upon binding to biomolecules [22], [23]. The photostability of squaraine dyes is comparable to those of conventional cyanine dyes [23], but can be substantially increased by the synthesis of a squaraine-rotaxane [24], an interlocked structure wherein a macrocycle encases the electrophilic squarylium core, preventing its exposure to nucleophilic attack in solution (Fig. 1a).

**Figure 2:**
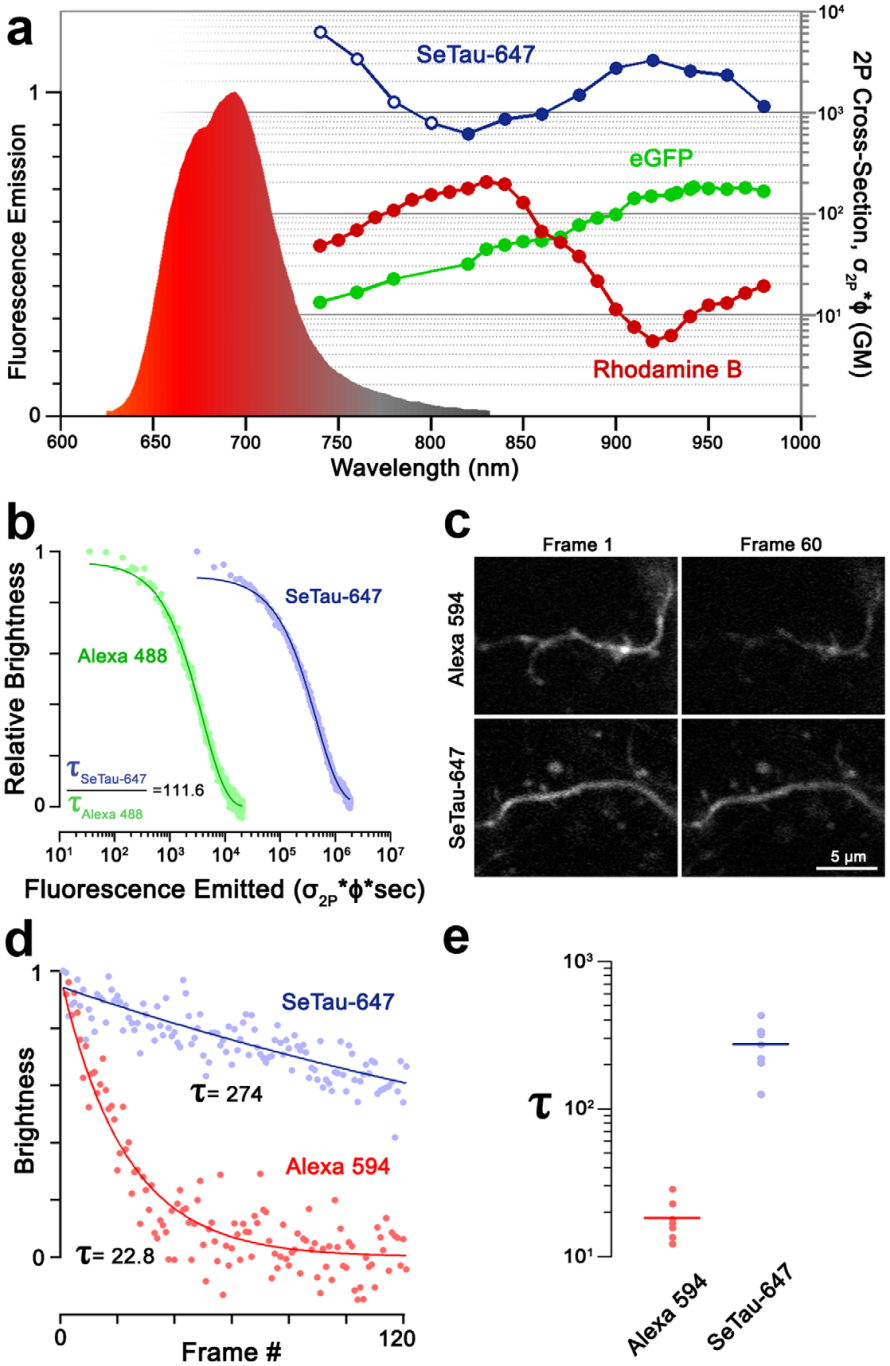
Brightness and photostability of the squaraine-rotaxane SeTau-647 measured *in vitro* and *in vivo*. **a)** Fluorescence emission spectrum of SeTau-647 (shaded curve) and two-photon action cross-section of SeTau-647, compared to published cross-sections [30] of a bright organic dye (Rhodamine B, red) and fluorescent protein (eGFP, green). For SeTau-647, data in closed circles were obtained by ratiometric fluorescence measurement, and data in open circles were obtained by Z-scan, which is less sensitive to single-photon phenomena. **b)** Simultaneous two-photon photobleaching of SeTau-647 and Alexa 488 at 920 nm in water. Dashed lines are fitted mono-exponential decay curves. The abscissa is the product of illumination time with the two-photon action cross-section of each dye, proportional to number of photons emitted per dye molecule. **c-e)** Photobleaching of SeTau-647 and Alexa 594 dextran conjugates in neuronal dendrites *in vivo*. **c)** Neurons were electroporated with 1 mM Alexa 594- (***top***) or SeTau-647- *(* ***bottom*** *)* dextran, and distal dendritic segments were imaged at the minimum laser intensity which allowed clear visualization. The 1^st^ (***left***) and 60^th^ (***right***) consecutive images are shown. **d)** Timecourse of photobleaching in example dendritic segments loaded with Alexa 594 (*red*) or SeTau-647 (*blue*) indicating. Lines are fitted monoexponential decay curves. Intensities have been normalized to the maximum and minimum of the fit curve. **e)** Distribution of fitted photobleaching rate (τ) for neurons loaded with Alexa 594 or SeTau-647. Each data point is the median fit bleaching rate over all dendritic segments imaged in one neuron. Horizontal bars denote the mean of each distribution.

Despite their unique optical properties [25], squaraines and squaraine-rotaxanes have yet to be applied to biological TPM, and little is known about their photochemical stability, toxicity, or two-photon fluorescence properties in living tissue. To address these questions, we screened a library of commercially available squaraine derivatives for strong excitation in the NIR window. We identified several dyes with spectra well suited for *in vivo* TPM, with action cross-sections in excess of 10,000GM (Fig. 1b). One promising candidate is the squaraine-rotaxane SeTau-647 (K9-4142), having a large two-photon cross-section across the NIR window, exceeding 6,000GM at 740 nm, with a second broad excitation maximum of 3,000GM at 920 nm (Fig. 2a). Such a wide absorption spectrum facilitates multiplex imaging with other fluorophores using a single excitation laser. Many fluorescent dyes show strongest two-photon absorption at twice the wavelength of their one-photon absorption peak. This is predicted for dyes that lack symmetry for which the absorption induces significant polarization [26], but this pattern can be violated in strongly absorbing dyes [15], and squaraines [16] show two-photon absorption strongly blue-shifted relative to twice their one-photon excitation peak. This shift allows imaging at lower excitation wavelengths, improving resolution and facilitating co-excitation with spectrally distinct fluorophores. For example, the 920 nm excitation peak of SeTau-647 overlaps peaks in two-photon excitation of eGFP and Alexa Fluor 488, but their emission spectra are well separated.

To be suitable for *in vivo* imaging, a dye should be stable and unreactive in the intracellular environment, and resistant to photobleaching. We determined the stability of SeTau-647 under two-photon excitation by simultaneously photobleaching SeTau-647 and Alexa Fluor 488 and measuring the time constant of decay in fluorescence for each dye. Despite showing a much higher cross-section, and thus undergoing a larger number of fluorescence transitions per molecule, SeTau-647 exhibits a longer photobleaching time constant than Alexa Fluor 488 under the same illumination conditions. We calculate that, on average, a SeTau-647 molecule emits 110 times as many photons as an Alexa Fluor 488 molecule prior to bleaching (Fig. 2b). Next, to determine photostability in the intracellular environment, we produced a dextran conjugate of SeTau-647 and introduced the dye into single neurons in the brain of *Xenopus laevis* tadpoles by single-cell electroporation [27]. To compare the performance of SeTau-647 to a commonly used fluorophore under typical experimental conditions, we electroporated individual neurons using the same concentration of dextran-conjugated SeTau-647 or Alexa Fluor 594 and performed *in vivo* imaging at the minimum laser intensity that allowed clear visualization of distal dendritic segments (Fig. 2c). SeTau-647 labelled neurons were clearly imaged using laser powers 4 to 8 times lower than those labelled with Alexa Fluor 594 (10 mW vs. 40–80 mW at the objective). Under these conditions, bleaching rates for Alexa Fluor 594 were over 10 times faster compared to SeTau-647 (Fig. 2c–e).

**Figure 3:**
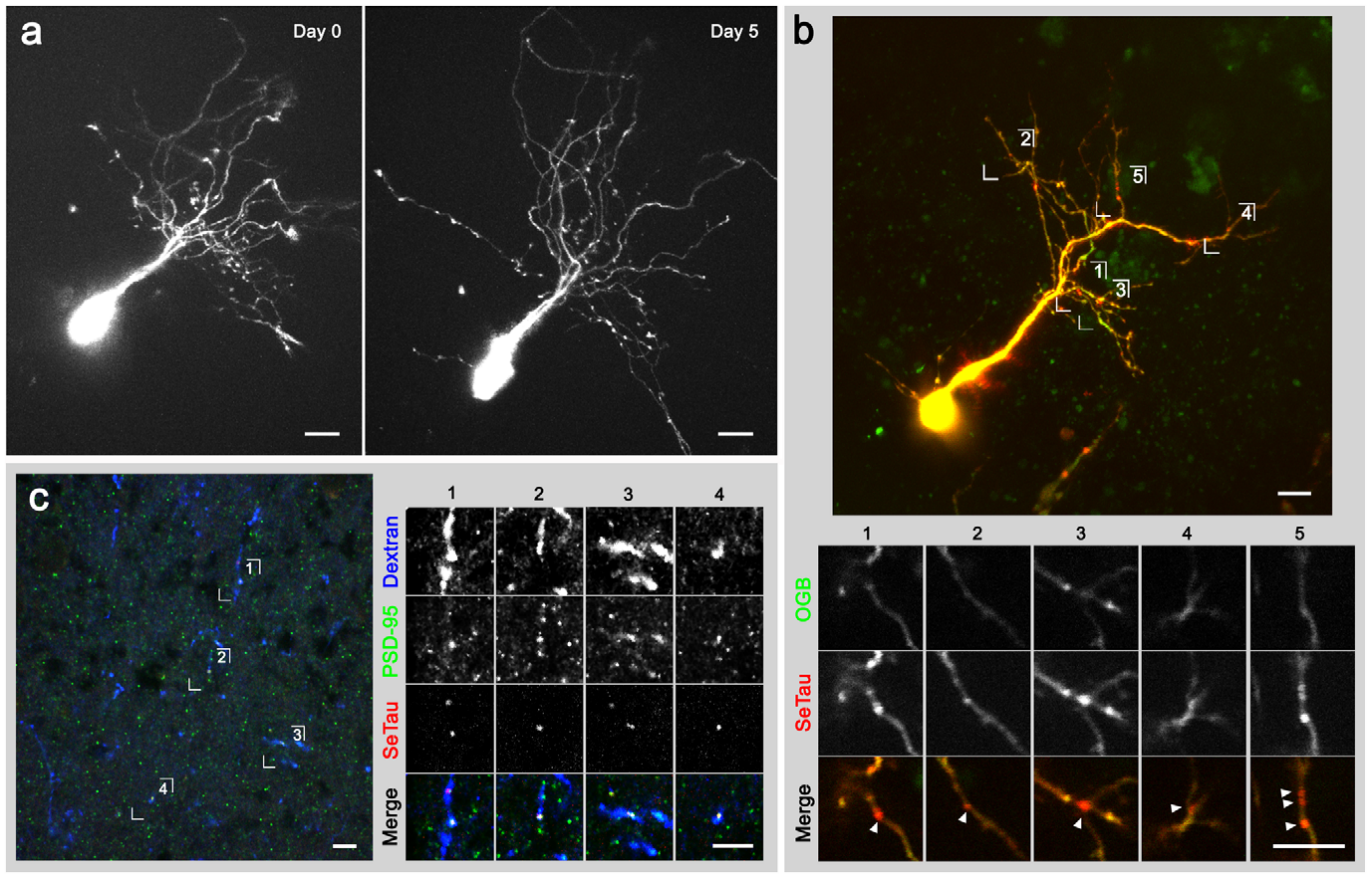
Cellular and subcellular labelling with SeTau-647 *in vivo*. **a)** Z-projection images acquired 3 hours (***left***) and 5 days (***right***) after single-cell electroporation with SeTau-647-dextran. Images were acquired at an excitation power of 10 mW at the back aperture of the objective (<8 mW at the focal plane). **b)** Targeted electroporation of a squaraine-tagged PDZ binding peptide labels postsynaptic density protein PSD-95. *(* ***top*** *)* Z-projection of a neuron filled with OGB-1 (*green*) and the squaraine-tagged peptide (*red*). *(* ***bottom*** *)* Expanded views of dendritic regions numbered above show punctate labelling of SeTau-647 (arrows). **c)** Anti-PSD-95 puncta colocalize with SeTau puncta in labelled tectal neuron dendrites. *(* ***left*** *)* Anti-PSD-95 immunostaining *(green)* of brain section containing dendrites of neurons labelled with SeTau PDZ-binding peptide *(red)* and Cascade Blue dextran *(blue)* as a space filler. *(* ***right*** *)* Expanded views of dendritic regions numbered at left. Scale bars: 10 µm.

To determine the long term intracellular stability of SeTau-647, we imaged dextran-conjugated SeTau-647 in neurons *in vivo* over a period of 5 days (Fig. 3a). All neurons imaged on the day of electroporation were present when imaged 5 days later (7/7, 100%). Neurons were imaged at the same laser intensity on both days and remained extremely bright 5 days after electroporation, with no signs of toxicity. Labelled neurons continued to grow and elaborate dendritic arbors (Fig. S1), suggesting that SeTau-647 does not interfere with cellular functions, and is stable over long periods in the intracellular environment.

A major benefit of a small, bright fluorophore is the ability to label discrete structures with low amounts of dye. To demonstrate such an application, we developed a fluorescent probe for PSD-95, a postsynaptic protein used as an indicator of excitatory synapses. While many excitatory synapses form at dendritic spines, synapses can also form without overt morphological correlates, and many neurons are non-spiny. A label that acutely identifies synapses would complement existing techniques relying on fusion protein overexpression, and facilitate imaging studies of synaptic physiology [10]. To acutely label endogenous PSD-95 in individual cells *in vivo*, we synthesized a peptide with high affinity for PDZ domain 3 of PSD-95 [28], fused to SeTau-647. When labelling endogenous proteins, target sites are limited and label concentration must be much lower than the peak concentration of the target. Optimal signal-to-noise ratios are achieved at a peptide concentration near the K_d_ of the peptide-target interaction. Imaging of small amounts of peptide bound at limited target sites benefits greatly from the higher gain provided by a brighter fluorophore.

To introduce labelled peptide into cells, we used two-photon guided single-cell electroporation [29] of tectal neurons, which allows targeting of individual neurons and observation of label diffusion after electroporation. Bright fluorescent puncta were distinguishable in neuronal dendrites within 5 minutes of electroporation, consistent with synaptic labelling (Fig. 3b,c). Each neuron was co-loaded with a green dye (Oregon Green BAPTA-1) to distinguish puncta from volume effects. Puncta could be clearly imaged at low excitation laser powers (<20 mW). Anti-PSD-95 immunostaining confirms that puncta coincide with endogenous PSD-95 (Fig. 3c). Nearly all (69/74; 93%) SeTau-647 puncta in the synaptic neuropil of the tectum overlapped with anti-PSD95 puncta, suggesting specificity of this label to PSD-95–containing compartments of the cell. A large proportion (69/97; 71%) of anti-PSD-95 puncta within labelled cells in the neuropil overlapped with SeTau-647 puncta. Some anti-PSD-95 puncta not showing SeTau-647 labelling may belong to other neurons, but overlap with labelled cells due to resolution limits. These results suggest that the PDZ-binding peptide acts as a specific and possibly sensitive marker for excitatory synapses in labelled neurons. Co-loading of a synaptic marker peptide and a fluorescent calcium indicator as described here may prove useful for identifying and tracking excitatory synaptic activity [10].

## Discussion

The squaraine dyes offer unprecedented fluorescence properties ideal for *in vivo* two-photon imaging. Squaraines have two-photon cross-sections and photostability approaching those of quantum dots, at molecular weights similar to other organic dyes, allowing labelling of subcellular structures, such as synapses, at low concentrations and with reduced functional impact. Critically, we demonstrate that squaraine derivatives are non-toxic, allowing long-term neuronal imaging. The demonstrated brightness and stability of these dyes promise to extend the limits of fluorophore concentration, imaging rate, illumination depth, and imaging duration for *in vivo* two-photon microscopy.

## Materials and Methods

### Fluorescent Labels

All squaraine and squaraine-rotaxane labels including SeTau 647 (K9-4142) are commercially available from SETA BioMedicals, Urbana IL. Dextran- and peptide- conjugates of SeTau-647 were synthesized from the dye NHS-ester as described below.

### Animal Rearing Conditions

Freely-swimming albino *Xenopus laevis* tadpoles were reared in 0.1× Steinberg’s solution (1× Steinberg’s in mM: 10 HEPES, 58 NaCl, 0.67 KCl, 0.34Ca(NO_3_)_2_, 0.83 MgSO_4_, pH 7.4) and housed at room temperature on a 12 hr light/dark cycle. Experiments were conducted with Stage 48 tadpoles in accordance with the Canadian Council on Animal Care guidelines, and were approved by the Animal Care Committee of the University of British Columbia Faculty of Medicine.

### Two-photon Action Cross-sections

Two-photon cross-section measurements and *in vivo* imaging were performed on a custom-built two-photon microscope adapted from an Olympus FV300 confocal microscope (Olympus, Center Valley, PA) and a Chameleon XR Ti:Sapphire laser (Coherent, Santa Clara, CA). Two-photon cross-sections were determined by two-channel ratiometric fluorescence intensity measurement with Alexa Fluor 488 hydrazide (Invitrogen, A10436) and the open aperture Z-scan method, at wavelengths between 760–980 nm in 20 nm increments. For ratiometric intensity measurements (Figs 1b, 2a), the laser was scanned over a 100×100 µm region at a depth of 5 µm in a well containing the two dyes at 5 µM concentration. Z-scan transmission measurements were made in a 1 mm path length of 10 µM SeTau 647, using a silicon detector (10D, UDT Sensors, Hawthorne, CA). Fluorescence emission for ratiometric measurements was collected through the following optics: 1.1 NA water-immersion objective (Olympus LUMFLN 60× W), 700 nm dichroic (700dcxr), 700 nm shortpass filter (ET700sp-2p), 585 nm longpass dichroic (ET585/40 m; Ch1 transmitted, Ch2 reflected), 610 nm longpass filter (E610LPv2, Ch1 only), 500–550 nm bandpass filter (HQ525/50M, Ch2 only). All filters were purchased from Chroma Technology, VT. We performed simultaneous current measurements on the two detectors channels while imaging the mixture of dyes. We subtracted from these currents the current measured with water alone at the same excitation wavelength. The subtracted currents were converted to brightnesses for each channel by compensating for the known parameters of the microscope/sample system: the emission spectra of the two dyes, the transmission spectrum of the objective, dichroics, and emission filters, and the gain and sensitivity spectra of the detectors. We performed linear unmixing to convert the channel intensities to brightnesses for each dye, using empirically measured bleedthrough of each dye on our microscope system. Bleedthrough was lower than 1% in all cases. Two-photon action cross-sections were then obtained by multiplying the ratio of brightnesses of the two dyes by the known action cross-section of Alexa Fluor 488 hydrazide. To confirm accuracy of cross-section measurements, we measured the cross-section of Alexa Fluor 594 using the same methods, obtaining values within 15% of those previously reported. Below 800nm, single-photon fluorescence and scattering become non-negligible in some bulk dyes, interfering with ratiometric measurement of cross-sections. At these wavelengths, we used the Z-scan method to measure and fit two-photon absorption, and single-photon fluorescence quantum yield to infer action cross-sections (Fig. 2a), which agreed well with those measured by the ratiometric method. Previously published cross-sections [30] were obtained from the Developmental Resource for Optical Imaging Electronics (DRBIO, www.drbio.cornell.edu).

### Labelled Dextran Synthesis and Single-cell Electroporation

SeTau-647-dextran was synthesized via coupling of the dye-NHS-ester with equimolar amounts of a 10 kDa amino dextran (Invitrogen, D-1860) in pH 8.5 borate buffer, and subsequent purification using a 6 kDa MWCO desalting column (Bio-Rad, 732–6228). For *in vivo* photobleaching and multi-day imaging experiments, individual neurons within the optic tectum of the intact tadpole brain were filled with SeTau-647 or Alexa Fluor 594 dextran (D-22913) using single-cell electroporation [27]. Tadpoles were briefly anaesthetized with 0.02% 3-aminobenzoic acid ethyl ester (MS222, Sigma). A sharp glass pipette (∼0.6 µm tip diameter) filled with dye dissolved in ultrapure water was inserted into the cell body layer of the tectum, and electroporation was performed using an Axoporator 800A (Molecular Devices, Sunnyvale, CA; stimulus parameters: pulse intensity = 1 µA; pulse duration = 300 µsec; pulse frequency = 900 Hz; train duration 14 msec).

### *In vivo* Imaging and Photobleaching

*In vitro* photobleaching was performed at a depth of 5 µm in a well (2 mm diameter) containing 0.5 mM SeTau-647 and Alexa Fluor 488 hydrazide, using the same detection setup described above. Intensity traces were obtained while scanning the excitation laser across a square region (∼100×100 µm) at 500 ms/frame. Resulting traces were fit with an exponential decay curve and normalized to the maximum intensity and minimum of the fit. For *in vivo* photobleaching experiments (Fig. 2c–e) tadpoles were reversibly paralyzed with 4 mM pancuronium dibromide prior to imaging, and placed in an imaging chamber perfused with oxygenated Steinberg’s solution throughout imaging. Neurons were electroporated using 1 mM-equivalent concentrations of Alexa 594- or SeTau-647-dextran. These were concentrations of dextrans with absorption at 600 nm equal to that of 1 mM free dye. Alexa 594 and SeTau-647 were imaged at 800 nm and 920 nm, respectively. Distal regions of loaded neuronal dendrites were imaged at the minimum laser intensity, which allowed dendrite boundaries to be clearly visible in a single frame (256×256 pixels 34×34 µm, 500 ms/frame). Both dyes were imaged with the filter set for Ch1 described above. This filter set, combined with the quantum efficiency of the detector (Hamamatsu R4632), results in detection of 2% and 0.8% of Alexa 594 and SeTau-647 emission, respectively. For 5-day imaging experiments (Fig. 3a, S1), neurons were imaged on the day of electroporation (Day 0) and 5 days later (Day 5) using the same laser intensity (<8 mW at the sample). Image stacks were registered by automated rigid-body transformation and maximum intensity projections are shown. Neurons were drawn in 3D using our custom written software Dynamo for analysis of dendritic arbor morphology and growth (Fig. S1).

### Targeted Electroporation and PSD-95 Labelling

To label endogenous PSD-95, we used the peptide sequence RVRLQTSV, which has high affinity for PDZ domain 3 of PSD-95, as identified in a phage display screen [28]. This sequence was modified by addition of a triglycine linker and an arginine residue (RGGGRVRLQTSV), and N-terminal coupled with SeTau-647. Coupling was performed during synthesis before resin cleavage to improve separation of product from the similar-sized reagents. The labelled peptide was purified by high performance liquid chromatography on a C8 column after cleavage.

For two-photon guided single-cell electroporation, the tectum was loaded with Cascade Blue Dextran (Invitrogen, D1976) to visualize the tectum and identify mature cells for electroporation. This dye shows virtually no two-photon absorption above 900 nm, and does not interfere with subsequent cellular imaging. Targeted electroporation was performed at 830 nm excitation. The electroporation pipette, containing 1.8 mM Oregon Green Bapta-1 (Invitrogen, O6806) and 0.8 mM SeTau-labeled peptide was inserted into the tectum and placed against a single mature tectal cell. Each cell was electroporated with a single 10 ms square pulse of between 18–25V. Morphological imaging was performed at 920 nm excitation.

### Immunohistochemistry

Several neurons per brain were co-loaded with Cascade Blue Dextran and the SeTau-647 peptide label using single-cell electroporation. 30 minutes after loading, brains were extracted, fixed in BT fixative (4% paraformaldehyde, 4% sucrose 0.12 mM CaCl in 0.1 M PBS, pH 7.4), washed. Brains were mounted in OCT medium and cryostat sectioned at 10 um. PSD-95 was detected with a monoclonal primary antibody (clone 7E3-1B8, Millipore) and Alexa-488-coupled secondary (Invitrogen, A11001). Sections were imaged with a confocal microscope (Olympus FV1000). SeTau-647 was excited at 633 nm and the emission was detected at 650–750 nm. Alexa Fluor 488 was excited and imaged separately from Cascade Blue and SeTau647 to ensure no bleedthrough between channels. Puncta were counted from seven imaged sections containing labelled cell dendrites across 3 tadpoles. Alexa-488 puncta were considered to be within labelled cells if the entire punctum overlapped with dendritic staining.

## Supporting Information

Figure S1 **SeTau-647 dextran labelled neurons continue to grow and elaborate branches. a)** Maximum intensity projection images of a neuron loaded with SeTau 647, on the day of electroporation (left) and 5 days later (right). **b)** Tracings of 3D image stacks in **a**. Greyscale intensity indicates Z-position of traced processes. Black circles indicate neuron somata. **c)** Total dendritic branch length (TDBL), including filopodia, of 5 neurons traced as in **b,** on the day of electroporation with SeTau 647, and 5 days later. Scale bars: 20 µm.
(TIF)
