# Supplementary figures and images for: Ultra-Bright and -Stable Red and Near-Infrared Squaraine Fluorophores for In Vivo Two-Photon Imaging
Source: PLoS One. 2012 Dec 14;7(12):e51980. doi: 10.1371/journal.pone.0051980 (PMC3522634; doi:10.1371/journal.pone.0051980)

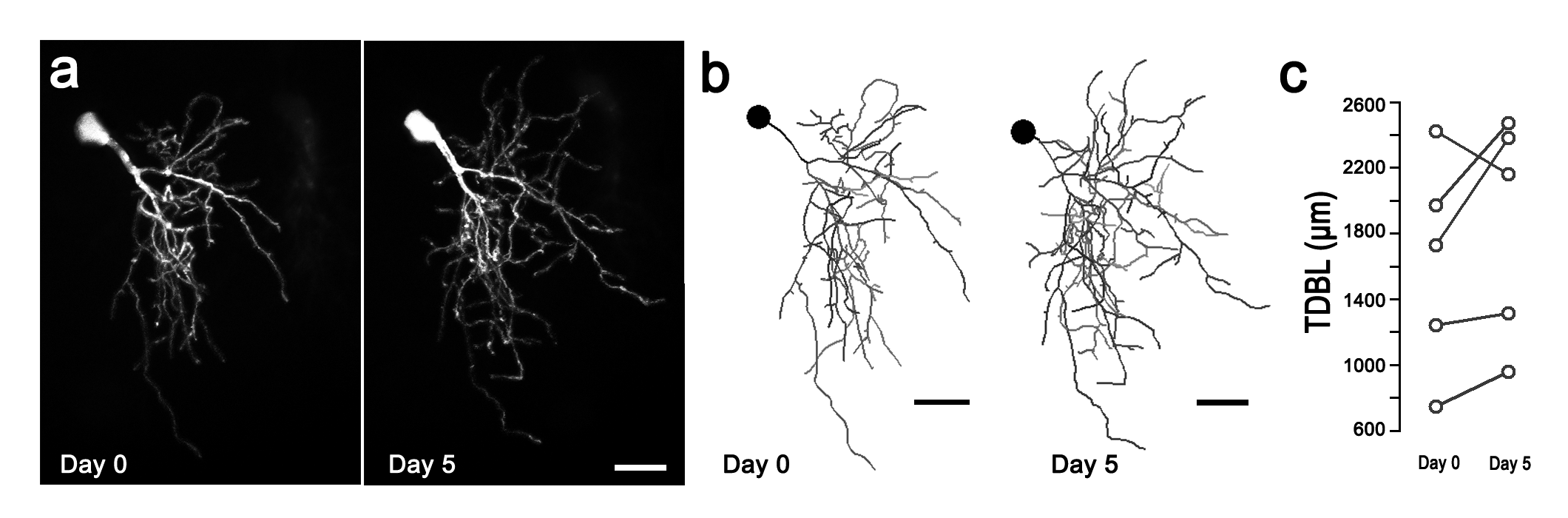

Supplement: Figure S1 — SeTau-647 dextran labelled neurons continue to grow and elaborate branches. a) Maximum intensity projection images of a neuron loaded with SeTau 647, on the day of electroporation (left) and 5 days later (right). b) Tracings of 3D image stacks in a. Greyscale intensity indicates Z-position of traced processes. Black circles indicate neuron somata. c) Total dendritic branch length (TDBL), including filopodia, of 5 neurons traced as in b, on the day of electroporation with SeTau 647, and 5 days later. Scale bars: 20 µm. (TIF) [file pone.0051980.s001.tif]
